# Differential Involvement of the Agranular *vs* Granular Insular Cortex in the Acquisition and Performance of Choice Behavior in a Rodent Gambling Task

**DOI:** 10.1038/npp.2015.133

**Published:** 2015-06-10

**Authors:** Abhiram Pushparaj, Aaron S Kim, Martin Musiol, Abraham Zangen, Zafiris J Daskalakis, Martin Zack, Catharine A Winstanley, Bernard Le Foll

**Affiliations:** 1 Translational Addiction Research Laboratory, Centre for Addiction and Mental Health, University of Toronto. Toronto, ON, Canada; 2 Department of Life Sciences, Ben-Gurion University of the Negev, Beersheba, Israel; 3 Campbell Family Mental Health Research Institute, Centre for Addiction and Mental Health, Toronto, ON, Canada; 4 Division of Brain and Therapeutics, Department of Psychiatry, University of Toronto, Toronto, ON, Canada; 5 Department of Pharmacology, University of Toronto, Toronto, ON, Canada; 6 Department of Psychology, University of British Columbia, Vancouver, BC, Canada; 7 Alcohol Research and Treatment Clinic, Addiction Medicine Services, Ambulatory Care and Structured Treatments, Centre for Addiction and Mental Health, Toronto, ON, Canada; 8 Department of Family and Community Medicine, University of Toronto, Toronto, ON, Canada; 9 Institute of Medical Sciences, University of Toronto, Toronto, ON, Canada

## Abstract

Substance-related and addictive disorders, in particular gambling disorder, are known to be associated with risky decision-making behavior. Several neuroimaging studies have identified the involvement of the insular cortex in decision-making under risk. However, the extent of this involvement remains unclear and the specific contributions of two distinct insular subregions, the rostral agranular (RAIC) and the caudal granular (CGIC), have yet to be examined. Animals were trained to perform a rat gambling task (rGT), in which subjects chose between four options that differed in the magnitude and probability of rewards and penalties. In order to address the roles of the RAIC and CGIC in established choice behavior, pharmacological inactivations of these two subregions via local infusions of GABA receptor agonists were performed following 30 rGT training sessions. The contribution made by the RAIC or CGIC to the acquisition of choice behavior was also determined by lesioning these areas before behavioral training. Inactivation of the RAIC, but not of the CGIC, shifted rats' preference toward options with greater reward frequency and lower punishment. Before rGT acquisition, lesions of the RAIC, but not the CGIC, likewise resulted in a higher preference for options with greater reward frequency and lower punishment, and this persisted throughout the 30 training sessions. Our results provide confirmation of the involvement of the RAIC in rGT choice behavior and suggest that the RAIC may mediate detrimental risky decision-making behavior, such as that associated with addiction and gambling disorder.

## Introduction

Gambling disorder is classified as an addictive disorder in the fifth edition of the Diagnostic and Statistical Manual of Mental Disorders-5, due to findings establishing its similarity to substance-use disorders in terms of clinical expression, brain origin, comorbidity, physiology, and treatment (APA, 2013). The insular cortex, or insula, is a brain area involved in interoception and thus numerous behaviors including conscious urges, anxiety, pain, cognition, mood, and substance abuse (Craig, 2002, 2009; Damasio *et al*, 2000; Goldman-Rakic, 1998; Hardy, 1985; Paulus and Stein, 2006; Watanabe *et al*, 1997). As such, it is not surprising that insular involvement has been identified in decision-making under risk and/or uncertainty (Clark, 2010). Importantly, the insula is composed of three distinct cytoarchitectural subregions ordered from the dorsal to ventral cortex, known as the granular, dysgranular, and agranular (Paxinos and Watson, 1986) and research has focused on the role of either the caudal granular (CGIC) or rostral agranular (RAIC) regions. Thus, the present study was undertaken to explore the differing roles of these two subregions in a rodent decision-making task.

Following the work of Naqvi *et al* (2007) in human smokers, our prior work identified the involvement of the CGIC in nicotine-taking and -seeking behavior using a rodent model (Forget *et al*, 2010; Pushparaj *et al*, 2013). The CGIC (bregma to −3.8 mm in rats) receives both general viscerosensory unimodal inputs (Cechetto and Saper, 1987) and nociceptive thalamic inputs (Gauriau and Bernard, 2004) along with somatosensory cortex inputs, while sending projections primarily back to the thalamus and somatosensory cortex along with projections to the caudate putamen (Shi and Cassell, 1998a). The CGIC is reciprocally connected to the RAIC primarily through intermediate relays in the dysgranular insular subregion (Shi and Cassell, 1998b). An important distinction between these two regions is that the entire granular insula, including the CGIC, is the only component of the insula that does not send projections to amygdalar nuclei (Shi and Cassell, 1998b). The RAIC sends projections to the ventral caudate putamen and the lateral nucleus accumbens (McGeorge and Faull, 1989; Shi and Cassell, 1998b), while having reciprocal connections with the basolateral amygdala (BLA) and the prelimbic cortex (Shi and Cassell, 1998b; Vertes, 2004). The RAIC is considered a high-order multimodal cortical region due to its inputs from the medial subdivision of the mediodorsal thalamic nucleus (Allen *et al*, 1991; Krettek and Price, 1977), which in itself is considered a high-order thalamic nucleus (Van der Werf *et al*, 2002), along with inputs from various medial thalamic nuclei thought to convey motivational/affective components of nociception. Others have confirmed the involvement of the CGIC (Contreras *et al*, 2007; Hollander *et al*, 2008; Scott and Hiroi, 2011) along with that of the RAIC, using various rodent models of addiction (Abdolahi *et al*, 2010; Contreras *et al*, 2012; Di Pietro *et al*, 2008; Seif *et al*, 2013).

Models of decision-making under risk are particularly useful for studying gambling disorder. Models of decision-making under expected uncertainty, or risk, involve overt presentation of specific probabilities and quantities for reward/loss, whereas models of unexpected uncertainty, or ambiguity, involve a clear understanding of the existence of risk, but not the specific probabilities/quantities. To our knowledge, only two studies have examined the insular cortex in a rodent model of decision-making under risk (Ishii *et al*, 2012; St Onge and Floresco, 2010), although a recent study has demonstrated that a dopamine D1 antagonist infused into the RAIC promotes impulsive decision-making (Pattij *et al*, 2014). Both studies examining decision-making under risk inactivated the RAIC, with one study finding no effect (St Onge and Floresco, 2010) and the other study finding a reduction in risk taking (Ishii *et al*, 2012).

The current study examines the involvement of two insular subregions, the RAIC and CGIC, in a rodent model of decision-making under risk, the rat gambling task (rGT), which is similar to the Iowa gambling task (IGT), a human model of decision-making under risk (Zeeb *et al*, 2009). The rGT shares a similar design and contingency structure with the IGT but differs from the IGT, as rodents are given a ‘forced-choice' training period to learn the contingencies for each of the four options (ie, reward amounts and punishment durations, along with probabilities). Human subjects in the IGT are presented with four decks of cards to choose from, but are never informed of the contingencies associated with each and must learn these through choosing from each deck. Thus, the rGT is a model of decision-making under risk, but not ambiguity. To differentiate between involvement in performance and acquisition of the task, we either inactivated the insula subregions, using local infusions of GABA receptor agonists, after 30 sessions of rGT training had established stable choice preference or lesioned them before any behavioral training. Owing to the reciprocal connectivity of the RAIC, but not the CGIC, with areas such as the BLA and orbitofrontal cortex (Shi and Cassell, 1998b; Vertes, 2004), previously determined to be involved in choice behavior on the rGT (Zeeb and Winstanley, 2011), we hypothesized that only the RAIC would be involved in both the acquisition and performance of the rGT.

## Materials and Methods

### Subjects

Male Long–Evans rats (Charles River, Lachine, QC) weighing 300–325 g at the start of experiments were maintained on ~20 g of rat chow daily and *ad libitum* water while in their home cages. Animals were single-housed in a temperature-controlled room on a 12-h reverse light cycle, with all behavioral testing occurring during the dark phase.

### Experimental Design

Experiments were conducted to examine the role of insular subregions (CGIC *vs* RAIC) at different rGT performance periods (acquisition of rGT choice behavior *vs* prior established rGT choice behavior). Acquisition experiments involved bilateral lesions of the RAIC or CGIC given before any behavioral training. Experiments examining established rGT performance involved bilateral cannulation of the RAIC or CGIC following acquisition of choice behavior (ie, animals experienced 30 days of daily rGT sessions).

### Surgery

Animals were anesthetized in an isoflurane (5%) induction chamber before being positioned in a stereotaxic apparatus (Kopf, Model 900) after which anesthesia was maintained using isoflurane (1%–2%) delivered via nose cone. The animals' heads were shaved and local anesthetic (0.1 ml bupivicaine, 0.125%) was injected at the incision site before Betadine was applied to clean the area. An incision was made along the midline and the skull exposed. Location of the bregma and lambda were determined and the skull was leveled. Sites of interest were determined relative to the bregma as follows, CGIC: anteroposterior −0.4 mm, lateral±4.8 mm; RAIC: anteroposterior +2.8 mm, lateral±4.0 mm. Small holes were drilled at the respective sites for each animal. For animals receiving bilateral cannulation, 22G stainless-steel guide cannulae (Plastics One, Roanoke, VA) were lowered relative to the dorsoventral coordinate taken from the cranial surface at the site of interest, which was as follows: CGIC: +5 mm, RAIC: +6.5 mm, with CGIC cannulae being implanted at a 10° divergent from the vertical. Guide cannulae were then fixed to the skull with screws and dental cement, and sealed with a stainless-steel occluder. For animals receiving bilateral lesions, a 28G stainless-steel injector, coupled by polyethylene tubing to a 10-μl Hamilton syringe in a microinfusion pump (Harvard Apparatus, Model 22, South Natick, MA), was slowly lowered relative to the dorsoventral coordinate taken from the cranial surface at the site of interest, which was as follows: CGIC: +6 mm, RAIC:+7.5 mm, with the CGIC injector again at a 10° divergent from the vertical. Over the course of 2 min, ibotenic acid (0.2 μl/side, 20 mg/ml in phosphate buffered saline) or vehicle was delivered to the site of interest and the injector was left in place for 5 min to allow for diffusion, and finally the incision was closed with wound clips. All animals were given prophylactic antibiotics (Derapen; 30 000 U, IM) and analgesic (Ketoprofen; 0.1 mg/kg, SC), and given a minimum of 1 week recovery in their home cages.

### Behavioral Equipment

Behavioral testing occurred in traditional five-choice serial reaction time task (5CSRTT) chambers (Med Associates, Roanoke, VA) controlled by software written in MED-PC running on an IBM-compatible computer (see Zeeb *et al.*, 2009 for further details). The chambers had five holes on one wall of the chamber, with a houselight above the holes, and a food receptacle in the middle of the opposing wall to which pellets (45 mg Dustless Precision Pellets, Bio-Serv, Flemington, NJ) could be delivered by an external dispenser. All five holes and the receptacle could be illuminated by a light contained within and nose-poke responses could be detected by infrared sensor. Only the outer four of the five holes were used in all experimental procedures (ie, the middle hole, 3 of 5, was unused).

### Pre-rGT Training

**Figure 1:**
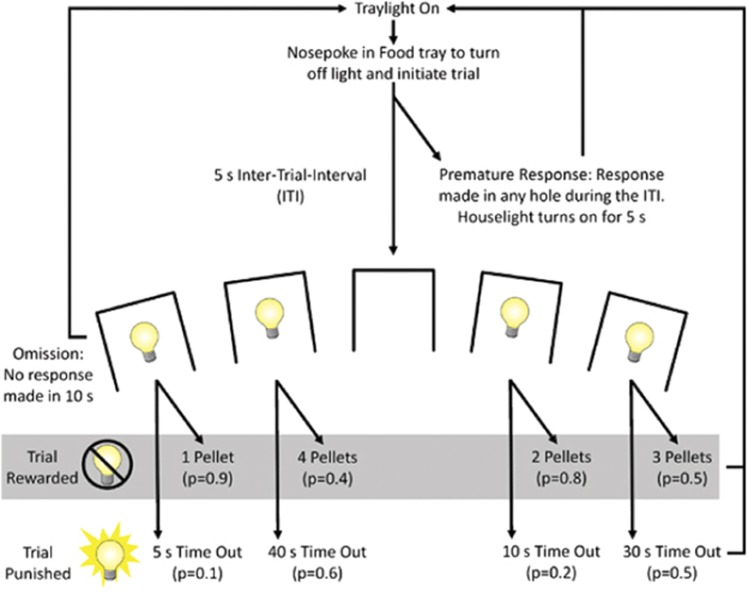
Trial structure of the rat gambling task (rGT). Number of pellets rewarded and duration of punishing timeout are stated for each option above their respective probabilities of occurrence. Assuming exclusive choice of an specific option throughout the session, its maximum number of pellets obtainable are stated at the bottom of the diagram, thus indicating optimal choice order of P2>P1>P3>P4. (Schematic taken with permission from Zeeb *et al*, 2009).

Animals were trained in 30 min daily sessions on a task similar to the 5CSRTT, with the exception of the middle hole (hole 3 of 5) being unused. Animals were trained to make a nose-poke response into the illuminated hole within 10 s, which resulted in a sucrose pellet being delivered to the food receptacle. The illuminated hole changed at each trial with equal number of presentations of each possible hole over the course of the session. Once all animals demonstrated two consecutive sessions where 100 trials were completed with 80% of trials being correct and <20% were omitted (ie, no response made within 10 s), the group experienced 12 daily sessions (30 min each) of a forced-choice version of the rGT where only one of the holes was illuminated at each time. This ensured that all animals had equal experience with the specific number of rewards (ie, 45 mg pellets) and specific duration of punishment (ie, time-out period), and respective probabilities of each, for the four different holes. Animals were counterbalanced on the forced-choice rGT with version A or version B, corresponding to which they would undergo in the subsequent rGT acquisition. Version A and B differed only based on the assignment of each contingency (P1–P4; see Figure 1 for contingencies) to its respective hole (Version A: hole 1=P1, hole 2=P4, hole 3=P2, and hole 4=P3; Version B: P4, P1, P3, and P2, respectively).

### Rat Gambling Task

The rGT has been previously described (Zeeb *et al*, 2009) and is outlined in Figure 1. Briefly, animals were given 30 daily sessions (30 min each) where they were required to make a nose-poke response into the illuminated food receptacle to start a trial. Once the response was made, the chamber was darkened for a 5-s intertrial interval (ITI) during which responses made were recorded as being premature responses and resulted in a 5-s houselight illumination and time-out period (during which responding had no effect) followed by an illumination of the food receptacle, which allowed the animal to begin another trial. If no premature response was made during the ITI, the four response holes were illuminated and the animals had 10 s to nosepoke into any of the four holes or else these lights were turned off and the food receptacle light was turned back on with the trial being counted as an omission. If the animal responded into one of the four holes before 10 s, the four lights were turned off and the trial was either rewarded (food receptacle illuminated and pellet(s) delivered according to contingency for chosen hole—see figure for contingencies; collection of reward resulted in initiation of new trial) or punished (stimulus light of chosen hole flashed at 0.5 Hz for a duration of time-out period, according to contingency of chosen hole—see figure for contingencies—following which food receptacle was illuminated allowing for new trial to be initiated).

### Cortical Inactivation

For experiments examining insular subregion involvement in established rGT choice behavior, animals were implanted with guide cannulae, as described above, after completing a total of 30 rGT sessions. Following surgical recovery, animals were given an additional 10 rGT sessions for reacquisition before testing began. Stability of choice, trials, and premature responding were statistically confirmed across the last three sessions (*P*>0.05, choice: two-way repeated-measures ANOVA for both choice X session and session; trials and premature responding: one-way repeated-measures ANOVAs for session). On testing days, injection cannulae were coupled to a 10-μl Hamilton syringe by a polyethylene tubing (inner diameter: 0.58 mm; Plastics One) filled with a GABA agonist mixture (muscimol: 0.03 nmol/side and baclofen: 0.3 nmol/side; Sigma-Aldrich, St Louis, MO) or the vehicle (sterile saline) and inserted into the guide cannula after removing the occluder. Rats were first habituated to the procedure of inserting the injection cannulae 1 day before testing. Over the course of 1 min, 0.5 μl of the GABA agonist mix (or saline) was injected into each side (driven by a microinfusion pump, Harvard Apparatus, Model 22), 5–10 min before the session. After the infusion, 1 min was allowed for diffusion, the injectors were removed, and occluders were replaced. Inactivation *vs* vehicle testing was counterbalanced and animals were given five rGT sessions between testing with stability being statistically confirmed for each respective group (ie, animals receiving inactivation first *vs* vehicle first).

### Data Analysis

All statistical analyses were conducted using SPSS for Windows. The main variables analyzed were percentage choice for each option (P1–P4) and percentage of optimal (P1+P2) choice. All choice data were arcsine transformed to control for the effect of a ceiling. Data from the experiments involving lesions were analyzed using repeated-measures ANOVAs with choice (four levels, P1–P4), and session as within-subject factors and group (lesion *vs* sham) as the between-subjects factor. Sham groups for the lesions were pooled after no statistically significant difference was found from an ANOVA conducted with choice, session, and sham region as factors. Data from the experiments involving cortical inactivation were analyzed using a two-way repeated-measures ANOVA with choice (four levels, P1–P4), and condition (inactivation *vs* vehicle) as within-subject factors and with group (optimal *vs* suboptimal) as a between-subject factor. Animals were defined as optimal if their responding for P1+P2 was >50% for the two sessions before each inactivation/vehicle session (average of four sessions total). If the outcome of the main ANOVA yielded significant effects (*P*<0.05), further *post-hoc* tests were performed.

### Histology

Following completion of behavioral testing, animals were given an overdose of sodium pentobarbital before brains were extracted and flash-frozen in liquid isopentane (kept at approximately −50 °C). Brains were stored at −80 °C before being sliced into coronal serial 30-μm sections throughout the respective subregion of interest and stained with cresyl violet. The extents of the lesions or the injector tips were mapped onto standardized sections of the rat brain (Paxinos and Watson, 1986).

## Results

### Cannulae Placement and Lesion Analysis

**Figure 2:**
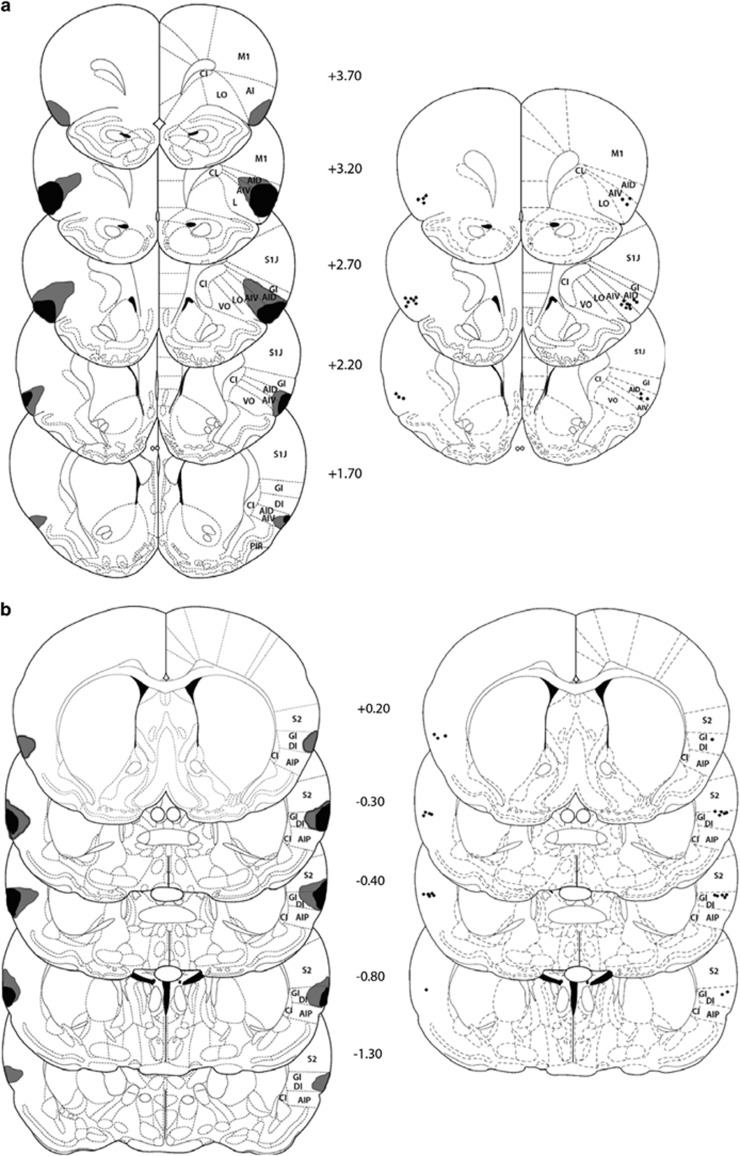
Location and extent of excitotoxic lesions or placements of injector tips. An illustration outlining the boundaries of the largest (gray) and smallest (black) area lesioned in any one section are shown for both the RAIC (a) and CGIC (b) lesion groups. Histological reconstruction of the injection sites in the RAIC (c) and CGIC (d), with black dots indicating locations of injector tips from animals included in statistical analysis. The number beside each reconstructed image indicates the distance (in millimeters) from the bregma. Schematic figure was published in Paxinos and Watson, 1986.

Illustrations of lesion location and extent along with those of cannulae placement are presented in Figure 2, whereas photomicrographs can be found in Supplementary Figure S2. Animals in the rGT acquisition experiments were excluded due to unilateral or overextended lesions; however, due to the difficulty of targeting specifically the granular or agranular regions without overlap, lesions were only considered incomplete if they did not cover <40% of the targeted area in the 15 brain slices anterior and posterior to the infusion site. Thus, a limitation is the sparing of the inferior layers of the target regions, in particular for the CGIC. Animals in the established rGT performance experiments were excluded due to incorrect cannulae placement or extensive damage surrounding the infusion site. It should be noted here, as a limitation, that all animals with CGIC lesions had some degree of damage extending into the dysgranular insula and secondary somatosensory cortex. Final numbers included in each experiment were as follows: lesions on acquisition of rGT: sham=32; RAIC=16; CGIC=14; inactivations on performance of established rGT: RAIC=14, CGIC=13.

### Effects of CGIC or RAIC Lesions on Pre-rGT Training

Animals were trained on a modified version of the 5CRTT, which only used four holes in order to correspond to the rGT. Lesioned animals, as compared with sham controls, appeared slower to acquire the behavior with a significantly lower number of trials initiated (Supplementary Figure S1A; session × lesion, RAIC *vs* sham: F_11, 506_=2.083, *P*<0.05; CGIC *vs* sham: F_11, 484_=2.196, *P*<0.05) and correct responses (Supplementary Figure S1B: session × lesion, RAIC *vs* sham: F_11, 506_=2.313, *P*<0.01; CGIC *vs* sham: F_11, 484_=3.014, *P*<0.001) and a significantly greater number of incorrect responses (Supplementary Figure S1C; session × lesion, RAIC *vs* sham: F_11, 506_=2.994, *P*<0.001; CGIC *vs* sham: F_11,484_=3.346, *P*<0.001). In addition, CGIC-, but not RAIC-, lesioned animals showed a significantly greater number of omissions compared with sham controls (Supplementary Figure S1D; session × lesion, RAIC *vs* sham: F_11, 506_=1.819, *P*>0.05; CGIC *vs* sham: session × lesion: F_11, 484_=3.590, *P*<0.001). However, by the last 3 days of this pre-rGT training phase, lesioned animals no longer differed significantly in responding from sham controls (for all variables: lesion, session × lesion, all Fs<2.29).

### Effects of CGIC or RAIC Lesions on Acquisition of the rGT

#### rGT decision-making

**Figure 3:**
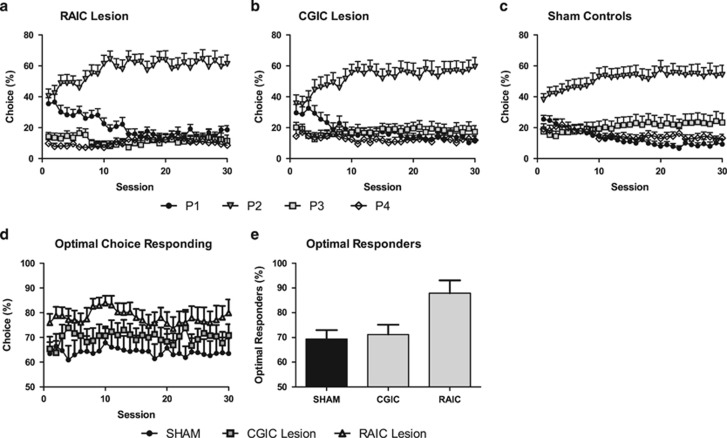
Effect of insular lesions on acquisition of the rat gambling task (rGT). Rostral agranular (RAIC-) (a), but not the caudal granular (CGIC-) (b), lesioned animals demonstrated significantly different acquisition of choice behavior as compared with sham controls (c). RAIC-, but not CGIC-, lesioned animals demonstrated a significantly greater preference for optimal choices (P1+P2) compared with sham controls (d). Finally, RAIC-, but not CGIC-, lesioned animals had a significantly greater percentage of optimal responders each session on average compared with sham controls (e).

The acquisition of choice behavior by RAIC, but not CGIC, lesioned animals significantly differed from that of sham controls (Figures 3a–c; session × choice × lesion, RAIC *vs* sham: F_87, 4002_=1.387, *P*<0.05; CGIC *vs* sham: F_87, 3828_=1.211, *P*>0.05).

RAIC, but not CGIC, lesioned rats showed a significantly greater preference for the optimal choices compared with sham controls (Figure 3d; lesion, RAIC *vs* sham: F_1, 46_=5.495, *P*=0.02; CGIC *vs* sham: F_1, 44_=2.666, *P*>0.05). Interestingly, there was no significant effect of session (session: F_29, 1711_=0.1914, *P*>0.05), suggesting overall optimal choice remained constant for the duration of training. This finding of consistent optimal choice (P1+P2) appears to contradict the observed change in choice pattern; however, this can be understood if a decrease in P1 choice over time is fully compensated for by a corresponding increase in P2 choice, while non-optimal choice remains fairly constant. Indeed this is the case here with significant effects of session observed for P1 (F_29, 1711_=1.743, *P*<0.01) and P2 choice (F_29, 1711_=1.631, *P*<0.05), although not for P3 (F_29, 1711_=0.233, *P*>0.05) or P4 (F_29, 1711_=0.346, *P*>0.05) choice. The average percentage of optimal animals for each session (made >50% optimal choice) in each group was compared for the entire acquisition period (Figure 3e). A significant effect of lesion was observed for percentage of optimal choice responders (F_2, 87_=124.2, *P*<0.001) with the RAIC-lesioned group having significantly greater optimal responders (87%) compared with CGIC-lesioned (71%) and sham groups (69% *P*<0.001).

Both RAIC- and CGIC-lesioned rats showed a significantly greater preference for P1 choice compared with sham controls (lesion, RAIC *vs* sham: F_1, 46_=6.354, *P*<0.05; CGIC *vs* sham: F_1, 44_=4.481, *P*<0.05). However, only RAIC-lesioned rats showed a significantly greater preference for P2 choice compared with sham controls (lesion, RAIC *vs* sham: F_1, 46_=4.479, *P*<0.05; CGIC *vs* sham: F_1, 44_=1.257, *P*>0.05). RAIC-, but not CGIC-, lesioned rats showed a significantly lower preference for P3 (lesion, RAIC *vs* sham: F_1, 46_=5.011, *P*<0.05; CGIC *vs* sham: F_1, 44_=2.245, *P*>0.05) and P4 choice compared with sham controls (lesion, RAIC *vs* sham: F_1, 46_=3.441, *P*>0.05; CGIC *vs* sham: F_1, 44_=1.545, *P*>0.05; session × lesion, RAIC *vs* sham: F_29, 1334_=1.529, *P*<0.05; CGIC *vs* sham: F_29, 1276_=0.969, *P*>0.05).

#### Other behavioral measures

Animals with lesions did not differ from sham controls in the level of premature responding, omissions, or perseverative responding observed during acquisition of the rGT (for all variables: lesion, session × lesion, all Fs<2.95) Supplementary Table S1. However, lesions of the CGIC, but not the RAIC, resulted in significantly increased latency to choose (lesion, RAIC *vs* sham: F_1,46_=3.854, *P*>0.05; CGIC *vs* sham: F_1, 44_=5.047, *P*<0.05) and collect reward (session × lesion, RAIC *vs* sham: F_29, 1334_=0.889, *P*>0.05; CGIC *vs* sham: F_29, 1276_=1.517, *P*<0.05) compared with sham controls along with a decreased number of trials initiated (lesion, RAIC *vs* sham: F_1, 46_=3.002, *P*>0.05; CGIC *vs* sham: F_1, 44_=5.883, *P*<0.05).

### Effects of CGIC or RAIC Inactivation on Established rGT Performance

#### rGT decision-making

**Figure 4:**
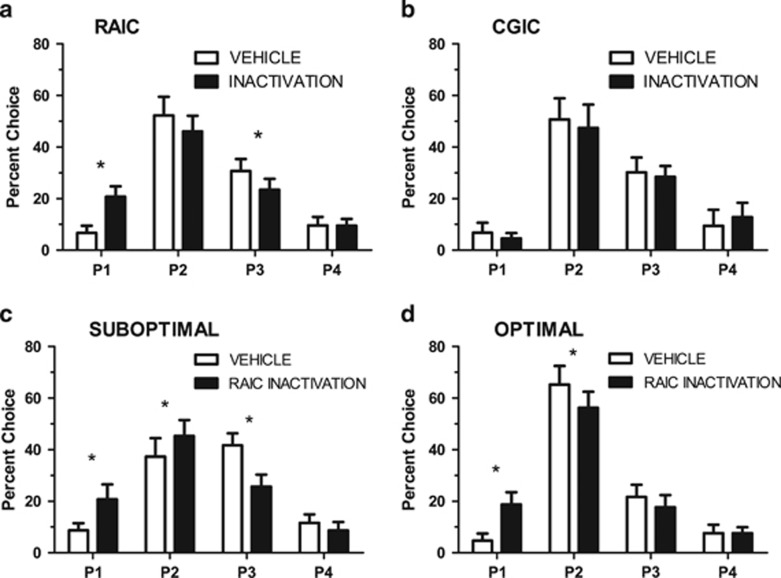
Effect of insular inactivations on established rat gambling task (rGT) performance. Inactivation of the rostral agranular (RAIC) (a) resulted in a significant increase in P1 choice and a significant decrease in P3 choice. Inactivation of the caudal granular (CGIC) had no significant effect (b). As only the RAIC inactivation showed a significant interaction effect with choice and optimal (>50% choice of P1+P2 during vehicle session) *vs* suboptimal group, these two subgroups were examined separately. For rats in the suboptimal group (c), RAIC inactivation resulted in a significant decrease in P3 choice and significant increases in P1 and P2 choice. Although for rats in the optimal group (d) RAIC inactivation resulted in a significant decrease in P2 choice and a significant increase in P1 choice. Data are expressed as the mean±s.e.m. with asterisks (*) indicating a significant difference (*P*<0.05) determined by paired *t*-tests.

Inactivation of the RAIC, but not the CGIC, significantly altered choice behavior (Figures 4a and b, respectively; inactivation × choice, RAIC: F_3,39_=3.683, *P*<0.05; CGIC: F_3, 36_=2.167, *P*<0.05). Specifically, RAIC inactivation resulted in a significant increase in P1 choice (*t*_13_=2.283, *P*<0.05) and a significant decrease in P3 choice (*t*_13_=1.912, *P*<0.05) with a decrease in P2 choice that did not reach significance (*t*_13_=1.583, *P*>0.05). Animals were defined as optimal if their responding for P1+P2 was >50% for the two sessions before each inactivation/vehicle session (average of four sessions in total). When animals were split into optimal *vs* suboptimal groups, only RAIC-inactivated animals showed a significant interaction effect (group × inactivation × choice, RAIC: F_3, 39_=4.323, *P*<0.05; CGIC: F_3,36_=1.954, *P*>0.05). For suboptimal animals (36% RAIC of group; Figure 4c) RAIC inactivation resulted in a significant decrease in P3 choice (*t*_5_=3.368, *P*<0.05) and a significant increase in P1 choice (*t*_5_=2.813, *P*<0.05) and P2 choice (*t*_5_=2.199, *P*<0.05). Interestingly, for optimal animals (64% of RAIC group, Figure 4d) RAIC inactivation resulted in a significant decrease in P2 choice (*t*_7_=3.006, *P*<0.05) and a significant increase in P1 choice (*t*_7_=4.259, *P*<0.01). Overall, RAIC inactivation resulted in the percentage of animals in the optimal group increasing from 64% to 79%. There were no suboptimal responders who became optimal responders following CGIC inactivation (optimal group remained at 62%).

#### Other behavioral measures

RAIC inactivation did not result in any significant changes in trials initiated, omissions, choice latency, collection latency, premature, or perseverative responses (all *t*'s<1.22, *P*>0.05) Supplementary Table S2. CGIC inactivation resulted in a significant increase in choice latency (*t*_12_=1.914, *P*<0.05) and a trend toward an increase in omissions (*t*_12_=1.581, *P*>0.05), whereas all other measures were not significantly affected (all *t*'s<1.28, *P*>0.05).

## Discussion

These results demonstrate insular involvement in decision-making under risk, as assessed by a rodent task with a contingency structure similar to the IGT. Subregion-specific involvement was demonstrated with RAIC disruption affecting both acquisition and performance of choice behavior, whereas CGIC disruption minimally affected acquisition. However, both RAIC and, to a greater extent, CGIC lesions affected the acquisition of appropriate responding on the pre-rGT serial reaction time task. Thus, it should be clearly noted that this suggests the insula is involved in the learning of a serial reaction time task, apart from its involvement in decision-making in the rGT. RAIC lesions resulted in acquisition of a consistently greater preference for ‘optimal' choices (options P1 and P2) which, when consistently chosen, produce the greater overall reward potential (295 and 411, respectively, *vs* 135 and 99 for P3 and P4, respectively). In rodents with established choice behavior, RAIC inactivation increased P1 choice and decreased P3 choice. Examining animals by subgroup of optimal (>50% choice of P1+P2) *vs* suboptimal RAIC inactivation increased P1 choice in both groups and decreased P3 choice in the suboptimal and optimal group. Interestingly, P2 choice decreased in the optimal group but increased in the suboptimal group following inactivation of the RAIC.

A limitation of the negative findings for the CGIC is the observation of increased latencies and decreased trials, in particular following lesions, suggesting potential motor impairments and/or decreased motivation to engage in the task. Although inactivations resulted in a slightly increased (10%) collect latency, our previous findings have demonstrated no effect of CGIC inactivations on pellets obtained or lever presses made for food self-administration under a progressive ratio schedule (Forget *et al*, 2010). However, the increased choice latency observed from both CGIC inactivations and lesions could be attributed to an increase in decision-making time. Interestingly, increased choice latency has also been observed following BLA inactivations in the rat cognitive effort task (Hosking *et al*, 2014) but not for BLA lesions on either the rGT (Zeeb and Winstanley, 2011), loss chasing, or betting tasks (Tremblay *et al*, 2014). Future work should examine whether this effect of increased choice latency following CGIC lesion/inactivation is also observed in other decision-making models.

To our knowledge, there are only two studies examining RAIC inactivation in rodent models of decision-making under risk with both examining performance not acquisition. St Onge and Floresco, 2010 demonstrated no effects of RAIC inactivation on a risk-discounting task, whereas the work of Ishii *et al* (2012) demonstrated that RAIC inactivation decreased risky (*vs* sure) choice and concluded that RAIC activity promotes risk-taking behavior. Owing to the nature of the rGT, our study cannot confirm this straightforward conclusion. Importantly, those prior two studies used models with only two options (risky *vs* sure). The optimal choice in the rGT is P2, which yields an average of 411 pellets per session when chosen exclusively. The other choices, in order of descending expected pellet yield if chosen exclusively, are P1 (295 pellets), P3 (135), and P4 (99). Importantly, this distribution of expected values results in overall choice percentage being high for P2, although individual animals may not prefer this option. Our results suggest that RAIC inactivation does not simply increase choice for the optimal risk-reward option. Both optimal and suboptimal animals appear to have an RAIC inactivation ‘shift' in choice behavior toward an increase in preference for options with higher probabilities of reward over punishment and lower number of pellets rewarded, but also lower durations of punishment. Unfortunately, due to the design of contingencies in our model (for a single trial, options with greater pellets rewarded=greater punishment duration=greater probability of punishment), we cannot distinguish whether this shift following RAIC inactivation is due to an increased preference for reward frequency and/or an increased avoidance of larger durations of punishment.

Although historically the insula was considered merely a primary gustatory cortex, more recent evidence has established it as an integrator and processor of somatosensory (Sterzer and Kleinschmidt, 2010), autonomic (Craig, 2002), and cognitive-affective information (Craig, 2009, 2010). According to the somatic-marker hypothesis, such an integral role in emotional feeling establishes this brain area as critical for guiding behavior (Craig, 2004; Damasio, 1996), in particular in situations involving risk and ambiguity (Craig, 2002; Naqvi *et al*, 2014; Singer *et al*, 2009). However, only the RAIC, not the CGIC, has been identified to have a large variety of bidirectional connections with critical subcortical and frontal cortical regions, such as the BLA and orbitofrontal cortex (Shi and Cassell, 1998b; Vertes, 2004), known to have a role in decision-making behavior (Zeeb and Winstanley, 2011). Thus, it is our belief, based on literature to be presented below, that RAIC activity represents an overall representation/valuation of the options available, along with the influence of contextual conditions (that is, said representation can be modulated by factors such as urgency, uncertainty and so on), and that this representation has a significant role in decision-making under risk.

Functional imaging in humans has demonstrated increased insular activity preceding risk-averse decisions (Kuhnen and Knutson, 2005), correlated with risk avoidance (Paulus *et al*, 2003), anticipation of risk (Preuschoff *et al*, 2006; Rudorf *et al*, 2012), monetary uncertainty (Critchley *et al*, 2001), and the interaction between urgency and expected values (Jones *et al*, 2011). Such data have supported a broader role for the insula in signalling aversive consequences *via* interoceptive signals (Paulus and Stein, 2006) and thus, it has been suggested that insular activity is required primarily for preventing disadvantageous risk (Clark *et al*, 2008). Yet another study has noted increased insular activity before a decision to take a risk and immediately after taking a risk (especially if risk resulted in a win), and decreased activity before refusing a gamble (Xue *et al*, 2010). As well, individuals with insular lesions have a lower propensity for risk in the rewarding ‘Gains' trials of the Cups Task (although no difference in propensity for risk in the punishing ‘Loss' trials) compared with healthy controls (Weller *et al*, 2009). Together, these results suggest that insular activity relays a contextualized interoceptive representation of available options during decision-making under risk, rather than merely identifying averse consequences of potential options. Additional support for this conclusion is the finding that individuals with insular cortex lesions do not demonstrate the ‘near-miss' effect (Clark *et al*, 2014), which is a greater motivation to have a slot-machine task after a loss that is close to a win (eg, a loss with two out of three stars), an effect that is reliably observed in healthy controls (Billieux *et al*, 2012; Clark *et al*, 2012; Clark *et al*, 2009). The same study demonstrated that individuals with insular lesions do not demonstrate ‘the gambler's fallacy,' which is the erroneous perception that recent consecutive outcomes are somehow less likely to occur even though events are known to be independent (eg, belief that ‘red is due to win now' on a roulette spin because multiple consecutive prior spins landed on black), also reliably observed in healthy controls (Ayton and Fischer, 2004). Individuals with insular lesions actually demonstrate a positive recency bias on the roulette task (ie, their choice of a color increases in likelihood as a function of the preceding run of that color), again suggesting an increased reliance on reward frequency (Clark *et al*, 2014).

Individuals with stroke-induced insular lesions have consistently demonstrated impaired decision-making behavior. This has been observed across multiple paradigms, including the IGT (Bar-On *et al*, 2003), the Cambridge Gambling Task (Clark *et al*, 2008), and the Cups Task (Weller *et al*, 2009). The latter two tasks involve decision-making under certain risk, with individuals choosing the value of a bet under differing risk in the Cambridge Gambling Task or choosing between a sure and a risky option in the Cups Task. On the other hand, the IGT involves decision-making under risk and uncertainty with choice between four options. The results of those studies are consistent in their demonstration that individuals with insular lesions lack risk adjustment, or the ability to adjust behavior with changing expected values. Our seemingly contradictory findings can be explained by the parametrical differences between previous studies and the rGT, and because unlike others, our model featured constant expected values. It should also be noted that any preference for reward frequency was likely not observed for individuals with insular lesions undertaking the IGT (Bar-On *et al*, 2003), because the original IGT used in that study had ‘stacked decks' with a greater probability of rewards occurring earlier. As such, overall impaired behavior of individuals with insular lesions on the IGT may have been due to an inability to adjust their choices toward the advantageous decks after having had initial experiences of large, consistent rewards in the disadvantageous decks. As well, greater reliance on reward frequency can explain the lower propensity of individuals with insular lesions to take risks in the rewarding ‘Gains' trials, while having no effect on their propensity for risk in the punishing ‘Loss' trials of the Cups Task (Weller *et al*, 2009). Overall, our results cannot confirm whether the RAIC and/or CGIC is the probable site of damage in individuals with stroke-induced insular lesions resulting in impaired risk adjustment, as our model used certain and stable probabilistic contingencies.

Based on the literature described above, one would conclude that both the valuations of reward (number of pellets) and punishment (time out duration) should be encompassed within an interoceptive representation held within the RAIC. Thus, assuming a reduction in RAIC function resulted in this interoceptive representation no longer affecting decision-making, one would assume that the relative valuation of reward/punishment amounts for each option no longer guided decision-making. This leaves the possibility that RAIC-manipulated animals instead increased their reliance on outcome probability (regardless of reward/punishment values) in their decision-making behavior. This may be a result of an increased reliance on the striatum in decision-making, as the ventral striatum is suggested to underlie learning associations between stimuli and responses *via* feedback, while the dorsal striatum mediates enacting decisions once those associations are established (Hiebert *et al*, 2014). Further work should be conducted to confirm whether RAIC manipulations specifically increase reliance on outcome probability, and whether this is mediated by the striatum. Importantly, this reliance on outcome probability does not suggest that lesions/inactivations of the RAIC improve overall performance in all cases, as rats in the optimal subgroup actually decreased choice of the best option, P2, with RAIC inactivations. As well, further work should be conducted under risk-free conditions, to verify that the results observed here are specific to decision-making under risk.

In conclusion, our results demonstrate that the RAIC, but not CGIC, is involved in decision-making behavior under risk in the rGT model. Taken along with other findings of insular involvement in the decision-making literature, we suggest that insular activity likely represents an overall interoceptive representation of the available options and thus increased insular activity can precede decisions of both risk avoidance and risk preference. In the case of gambling disorder, where such interoceptive representations likely contribute to detrimental risky decision-making behavior, manipulations of the RAIC may be of potential therapeutic benefit. Future work should examine the effects of manipulating insular activity, such as repetitive transcranial magnetic stimulation, on decision-making tasks and gambling behavior in human laboratory models.
