# Supplementary figures and images for: Differential Involvement of the Agranular vs Granular Insular Cortex in the Acquisition and Performance of Choice Behavior in a Rodent Gambling Task
Source: Neuropsychopharmacology. 2015 Jun 10;40(12):2832–42. doi: 10.1038/npp.2015.133 (PMC4864659; doi:10.1038/npp.2015.133)

**FIGURE S1**

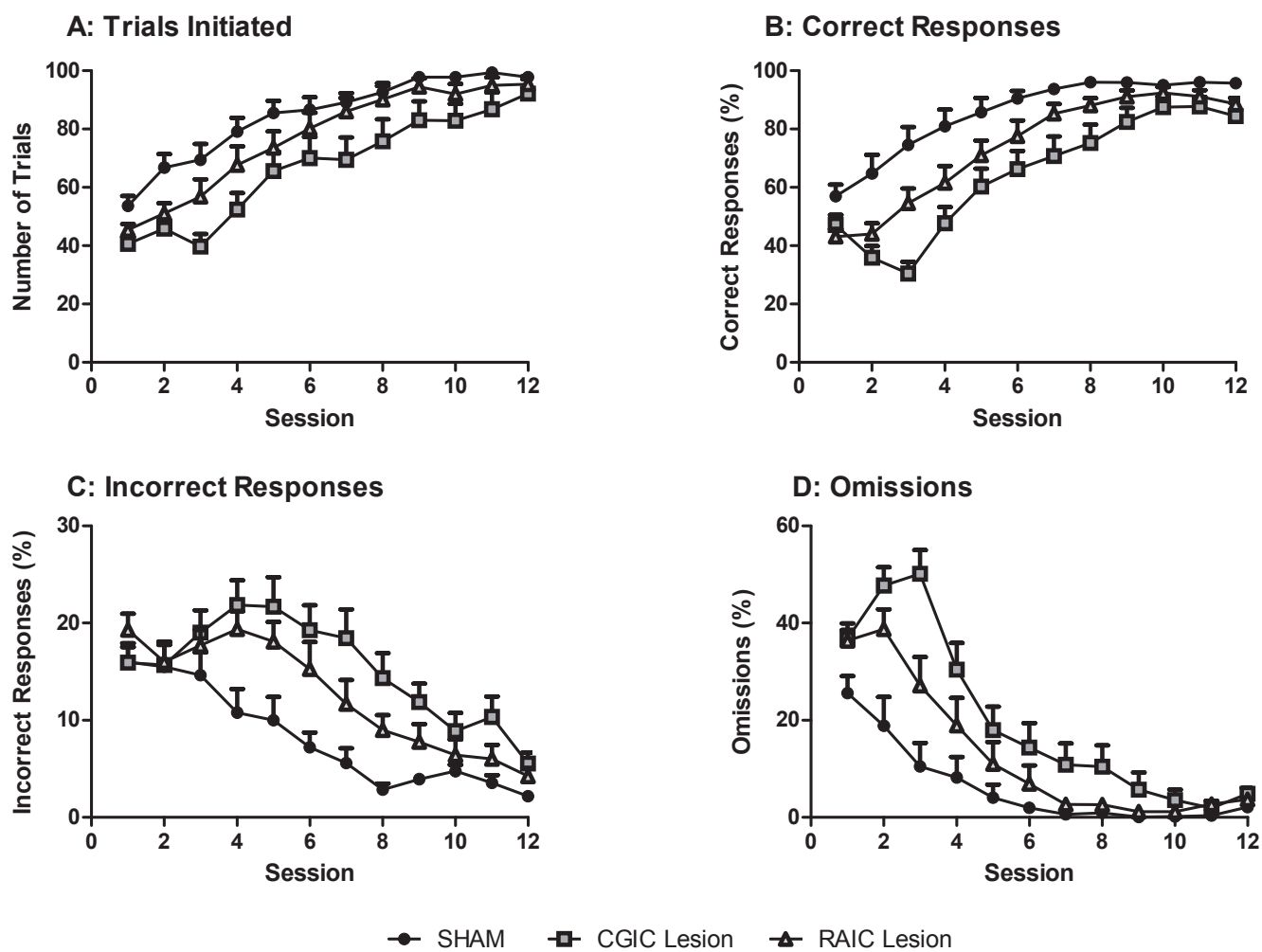

Supplement: Supplementary Figure 1 [file npp2015133x1.pdf]

FIGURE S2

**A**

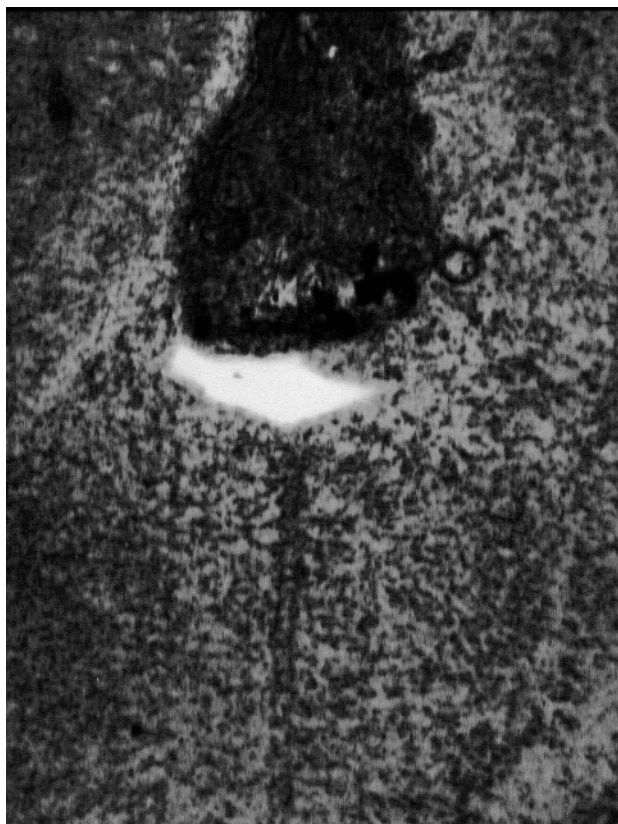

**B**

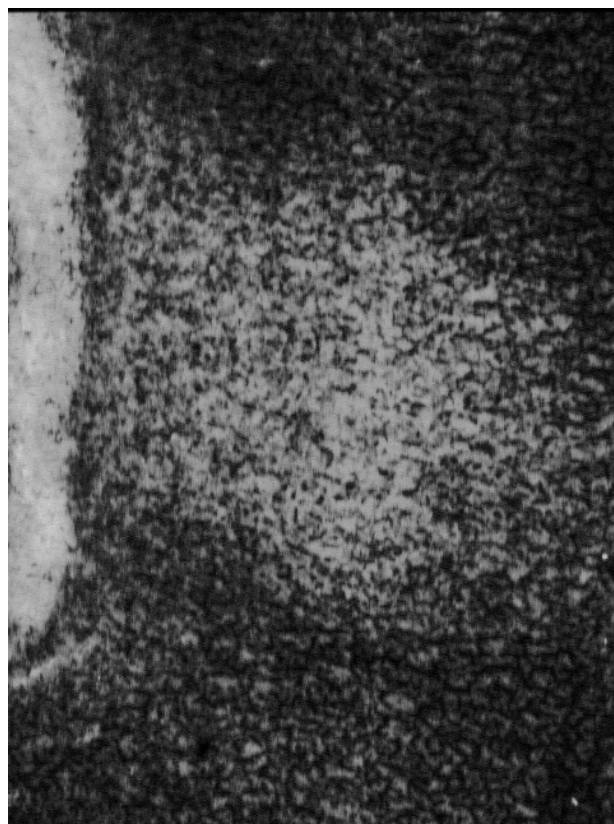

**C**

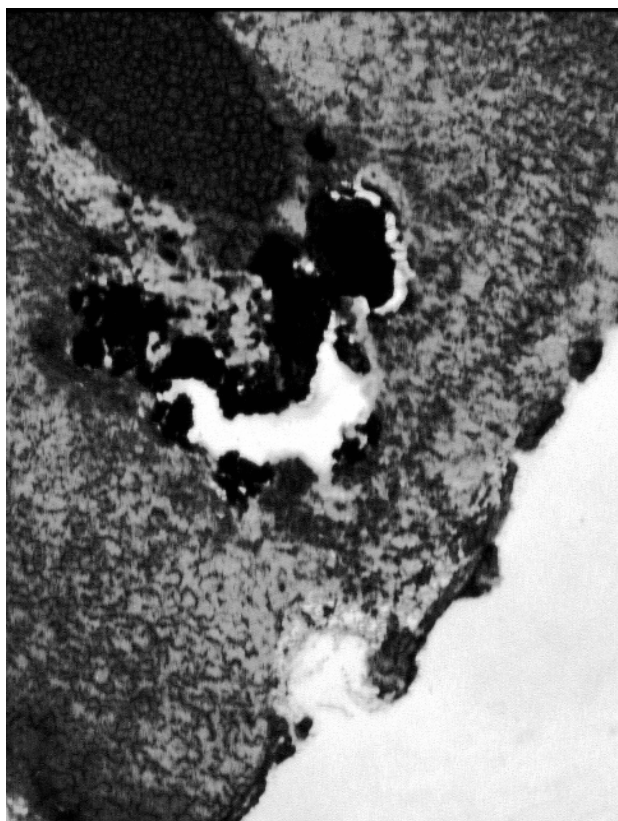

**D**

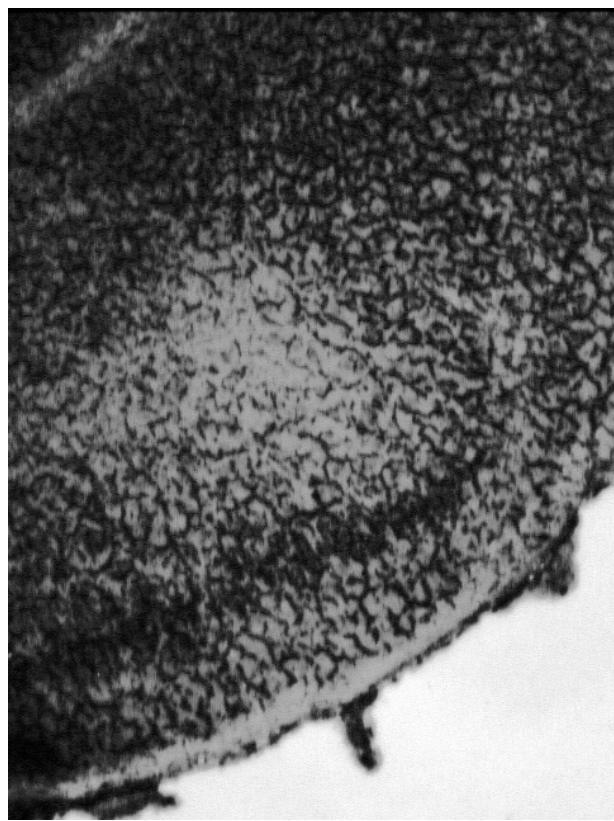

Supplement: Supplementary Figure 2 [file npp2015133x2.pdf]
