# Supplementary material for: Differential Involvement of the Agranular vs Granular Insular Cortex in the Acquisition and Performance of Choice Behavior in a Rodent Gambling Task
Source: Neuropsychopharmacology. 2015 Jun 10;40(12):2832–42. doi: 10.1038/npp.2015.133 (PMC4864659; doi:10.1038/npp.2015.133)
Supplement: Supplementary Figure Legends [file npp2015133x3.doc]

**Figure S1.** Effect of insular lesions on pre-rGT training. Animals with insular lesions showed a slower acquisition of response behavior on a modified version of the five choice serial reaction time task, as evidenced by an initial significantly lower number of trials initiated (A), significantly lower percentage of correct responses (B), significantly higher percentage of incorrect responses (C) and in CGIC, but not RAIC, lesioned animals a significantly higher percentage of omissions (D).

**Figure S2.** Representative photomicrographs of (A) CGIC cannulae placement, (B) CGIC lesion, (C) RAIC cannluae placement, and (D) RAIC lesion.
