# Supplementary material for: Differential Involvement of the Agranular vs Granular Insular Cortex in the Acquisition and Performance of Choice Behavior in a Rodent Gambling Task
Source: Neuropsychopharmacology. 2015 Jun 10;40(12):2832–42. doi: 10.1038/npp.2015.133 (PMC4864659; doi:10.1038/npp.2015.133)
Supplement: Supplementary Tables [file npp2015133x4.doc]

**SUPPLEMENTARY TABLES**

TABLE S1

|  | **Sham lesion** | **RAIC lesion** | **CGIC lesion** |
| --- | --- | --- | --- |
| **Trials initiated** | 112.92 ± 9.67 | 120.25 ± 12.32 | 87.67 ± 8.49 |
| **Omissions** | 1.94 ± 0.67 | 1.41 ± 0.44 | 2.27 ± 0.72 |
| **Choice latency** | 1.24 ± 0.22 | 1.16 ± 0.17 | 1.93 ± 0.29 |
| **Collection latency** | 0.94 ± 0.07 | 1.02 ± 0.09 | 1.33 ± 0.14 |
| **Premature responses** | 13.86 ± 3.41 | 12.06 ± 2.77 | 15.24 ± 3.38 |
| **Perseverative responses** | 114.57 ± 22.47 | 126.34 ± 19.46 | 130.79 ± 24.55 |

Data are expressed as the mean ± SEM for the last 5 sessions of rGT training. Omissions and premature responses are represented as a percentage. Latency values are in seconds.

**TABLE S2**

|  | **RAIC Veh** | **RAIC Inact** | **CGIC Veh** | **CGIC Inact** |
| --- | --- | --- | --- | --- |
| **Trials initiated** | 99.08 ± 8.97 | 105.24 ± 9.71 | 103.84 ± 10.59 | 95.28 ± 11.41 |
| **Omissions** | 1.31 ± 0.70 | 1.69 ± 0.63 | 1.29 ± 0.59 | 2.34 ± 0.81 |
| **Choice latency** | 1.38 ± 0.21 | 1.54 ± 0.17 | 1.43 ± 0.19 | 2.06 ± 0.29 |
| **Collection latency** | 1.06 ± 0.08 | 0.98 ± 0.10 | 1.11 ± 0.11 | 1.21 ± 0.14 |
| **Premature responses** | 15.24 ± 3.16 | 17.21 ± 2.74 | 14.52 ± 3.74 | 18.48 ± 3.49 |
| **Perseverative responses** | 134.15 ± 19.48 | 126.57 ± 21.46 | 127.84 ± 22.64 | 137.42 ± 18.77 |

Data are expressed as the mean ± SEM. Omissions and premature responses are represented as a percentage. Latency values are in seconds.
